# Drastic changes in ground-dwelling beetle communities following high-intensity deer culling: insights from an island ecosystem

**DOI:** 10.1093/ee/nvae013

**Published:** 2024-02-25

**Authors:** Blake M Dawson, Maldwyn J Evans, Philip S Barton, Masashi Soga, Kahoko Tochigi, Shinsuke Koike

**Affiliations:** School of Environmental and Rural Science, University of New England, Armidale, NSW 2350, Australia; Fenner School of Environment and Society, The Australian National University, Canberra, ACT, Australia; School of Life and Environmental Sciences, Deakin University, Geelong, Victoria 3216, Australia; Graduate School of Agricultural and Life Sciences, The University of Tokyo, 1-1-1 Yayoi, Bunkyo, Tokyo 113–8657, Japan; Institute of Global Innovation Research, Tokyo University of Agriculture and Technology, Fuchu, Tokyo 183-8509, Japan; Institute of Global Innovation Research, Tokyo University of Agriculture and Technology, Fuchu, Tokyo 183-8509, Japan

**Keywords:** ecosystem processes, necrobiome, Carabidae, Scarabaeidae, sika deer, Silphidae

## Abstract

The overabundance of large herbivores can have detrimental effects on the local environment due to overgrazing. Culling is a common management practice implemented globally that can effectively control herbivore populations and allow vegetation communities to recover. However, the broader indirect effects of culling large herbivores remain relatively unknown, particularly on insect species such as ground-dwelling beetles that perform key ecosystem processes such as decomposition. Here we undertook a preliminary investigation to determine how culling sika deer on an island in North Japan impacted ground-beetle community dynamics. We conducted pitfall trapping in July and September in 2012 (before culling) and again in 2019 (after culling). We compared beetle abundance and community composition within 4 beetle families (Carabidae, Scarabaeidae, Geotrupidae, and Silphidae), across seasons and culling treatments. We found each family responded differently to deer culling. Scarabaeidae displayed the greatest decline in abundance after culling. Silphidae also had reduced abundance but to a lesser extent compared to Scarabaeidae. Carabidae had both higher and lower abundance after culling, depending on the season. We found beetle community composition differed between culling and season, but seasonal variability was reduced after culling. Overall, the culling of large herbivores resulted in a reduction of ground-dwelling beetle populations, particularly necrophagous species dependent on dung and carrion for survival. Our preliminary research highlights the need for long-term and large-scale experiments to understand the indirect ecological implications of culling programs on ecosystem processes.

## Introduction

The overabundance of large herbivore species is a global problem (Côté et al. 2004, Gordon et al. 2004), which can cause detrimental effects on ecosystems (Barton et al. 2011, Gerhardt et al. 2013, Iida et al. 2016). For example, in Japan, deer overabundance has resulted in the loss of understorey vegetation and associated wildlife, soil erosion, and hindrance to forest regeneration (Côté et al. 2004), while in Australia the overabundance of kangaroos has had detrimental effects on plant communities (Gordon et al. 2021). Overabundant species can alter landscapes by consuming vegetation and degrading plant communities by limiting regrowth and dispersal processes (Côté et al. 2004, Velamazán et al. 2017). In some situations, overconsumption and selective foraging can alter the plant community composition and lead to local extinctions, as floral species that are not favored by the herbivore species gain a competitive edge (Akashi and Nakashizuka 1999, Gill and Beardall 2001, Gorchov et al. 2021). Moreover, the disturbance of plant communities by large herbivores can have cascading effects on habitat structure and its dependent fauna and food webs (Den Herder et al. 2004, Foster et al. 2014, Mahon et al. 2020). It can impact water, nutrient, and carbon cycling, and increase the susceptibility of the environment to exotic species invading (Eschtruth and Battles 2009, Beguin et al. 2011, Cardinal et al. 2012, Seki and Koganezawa 2013).

A common management practice to address herbivore overpopulation is to implement translocation or controlled culling programs (Putman et al. 2011, Driscoll et al. 2019, Ikeda et al. 2019). The goal of such programs is to reduce herbivore numbers to a desired level that reduces the ecological impacts of the species (Tanentzap et al. 2012, Enoki et al. 2016). Culling and associated permitted hunting also provides economic benefits when culled species are removed from the environment and used for human or pet food for commercial gain (Nugent and Choquenot 2004). Despite the often-negative perception of culling by the public (Sharp et al. 2011), research on the management practice has been shown to be ecologically effective and has been implemented globally, targeting a wide range of species such as deer (Wäber et al. 2013), boars (Croft et al. 2020), and kangaroos (Gordon et al. 2021).

While the negative effects of overabundant large herbivores are well established, there is a need to understand how the culling of herbivores might impact key ecological processes. If herbivore numbers are reduced, for example, any potential benefits that culling provides could be impacted (Bardgett and Wardle 2003, Foster et al. 2014). Decomposer and scavenger communities depend on the carcass remains of large herbivores as a food resource and breeding substrate, while other decomposer species depend on dung for survival (Nichols et al. 2008, Butterworth et al. 2022). These decomposers play a key role in maintaining biodiversity and ecosystem functions and services such as nutrient and energy cycling (Barton et al. 2013, Englmeier et al. 2022). The sudden removal of a large number of herbivore species from an ecosystem may have unintended negative consequences on dependent species, which in turn could have bottom-up effects on the food web and broader ecosystem function (Iida et al. 2016). Further research on the effects of culling of herbivores as a management tool is required to determine how ecosystems might respond.

Nakajima Island in Japan offers a unique opportunity to study the effects of herbivore culling on biodiversity and ecosystems. Since the introduction of sika deer (*Cervus nippon* Temminck) to the island, the understorey vegetation has been under increased pressure due to high deer density (Yama et al. 2019). In an effort to mitigate the negative ecological impacts of the deer population, a controlled culling program was implemented on the island resulting in a drastic reduction in deer numbers (Ikeda et al. 2019). The island presents an ideal study system for examining the indirect effects of deer culling on ground-dwelling beetle communities in a natural environment without the risk of reintroduction of deer.

In this study, we investigated how ground-dwelling carabid (Carabidae), dung (Scarabaeidae, Geotrupidae), and carrion (Silphidae) beetles responded to a reduction in deer population on Nakajima Island, Japan. We focused on these beetles because of their clear role in ecosystem processes as predators and scavengers, and are likely dependent, in part, on large herbivores as a food resource and breeding substrate. We discuss our results in light of the effects of deer culling on the beetle community and how this may indirectly influence key ecological processes. This research will help to provide insight into the broader impacts of culling programs on ecosystems and the potential consequences for dependent species.

## Materials and Methods

### Study Site

We conducted our study on Nakajima Island (42°36ʹ N, 140°51ʹ E), which is centrally located within Lake Toya, at an approximate distance of 4 km from the lake. The snow-free period on the island spans from April to November. The majority of the island is covered by natural, broad-leaved forests, which consist of species like *Acer pictum* Thunb. subsp. *momo* H. Ohashi and *Tilia japonica* Simonk var. *japonica* Miq. Sika deer were introduced to the island in 1957–1966 and have since experienced a rapid increase in population, reaching a peak of 50 deer/km^2^ in response to a prohibition of hunting and the absence of natural predators (Ikeda et al. 2013). A controlled culling program was implemented in 2012 to reduce deer numbers, resulting in a deer density of roughly 12 deer/km^2^ in 2019 (Kaji and Takeshita 2022). Further information about the island ecology and deer populations are detailed in Ikeda et al. (2015) and Kaji et al. (2005).

### Beetle Sampling

We conducted two rounds of ground beetle community sampling on the island in 2012 (prior to culling) and 2019 (after culling). To capture beetles when they were most active in this region, we conducted sampling twice in each sampling year, once in July and again in September. We established 30 sampling plots along a 3 km transect, spaced 100 m apart from each other (only 10 sites in July 2012). The transect ran through the center of the island from the east to the west coast. We deployed a single pitfall trap, baited with cattle dung and fermented milk to each plot. Dung and fermented milk were used as they are known to attract ground-dwelling beetles (Suttiprapan and Nakamura 2007). We constructed the pitfall traps using plastic containers (22.5 cm diameter, 26.6 cm depth) and plastic cups (8.3 cm diameter, 11.5 cm depth). To set a trap, we buried a plastic container filled with fermented milk into the ground, ensuring the rim was level with the ground. We hung the plastic cup containing dung inside the container using wires. We also placed a plastic roof over the trap to protect it from rain and leaves. We left the traps in place for a period of 3 days, after which we counted and collected all the beetles. We identified them as the highest taxonomic unit possible using morphological characteristics (Ueno et al. 1985).

### Data Analysis

To investigate the effect of deer culling on beetle populations and community composition, we performed 3 statistical analyses. First, we analyzed the response of the beetle community composition to deer culling and performed a permutational multivariate analysis of variance test (PERMANOVA) using the Vegan package (Oksanen et al. 2022) in R (R Core Team 2023). We used the interaction of month and culling as fixed effects, and site as a random effect. We used a Bray–Curtis dissimilarity matrix to calculate the distance matrices. To visualize the beetle communities, we plotted the ordination as a non-metric multidimensional scaling (NMDS) plot. We also calculated Pielou’s evenness for each combination of month and culling to identify changes in overall relative abundances using the Vegan package (Oksanen et al. 2022).

Second, we conducted generalized linear mixed modeling (GLMM) to determine how the interaction of month (July and September), culling (before culling and after culling), and family (Carabidae, Scarabaeidae, Geotrupidae, and Silphidae) influenced beetle abundance. To control the effects of repeated measures, we included the site as a random effect. We fit a GLMM using the glmmTMB package in R, assuming a Poisson error distribution and a log-link function (Brooks et al. 2017). We then conducted pairwise comparisons of the predicted variables using the emmeans package (Lenth 2023), and assessed model performance using the performance package (Lüdecke et al. 2021).

Finally, to understand how deer culling influenced individual beetle species, we conducted a Bayesian ordination and regression analysis using the Boral package (Hui 2016). We assumed a negative binomial distribution and 2 latent variables and used the same interaction and random effect as the PERMANOVA. We grouped the beetle species by family and plotted the X-coefficient estimates as effect sizes. We interpreted the effects as significant if their 95% credible intervals did not cross the zero-effect posterior median line. All statistical analyses were performed in R version 4.2.3 and the plots were created using the ggplot2 package (Wickham 2016).

## Results

**Fig. 1.**
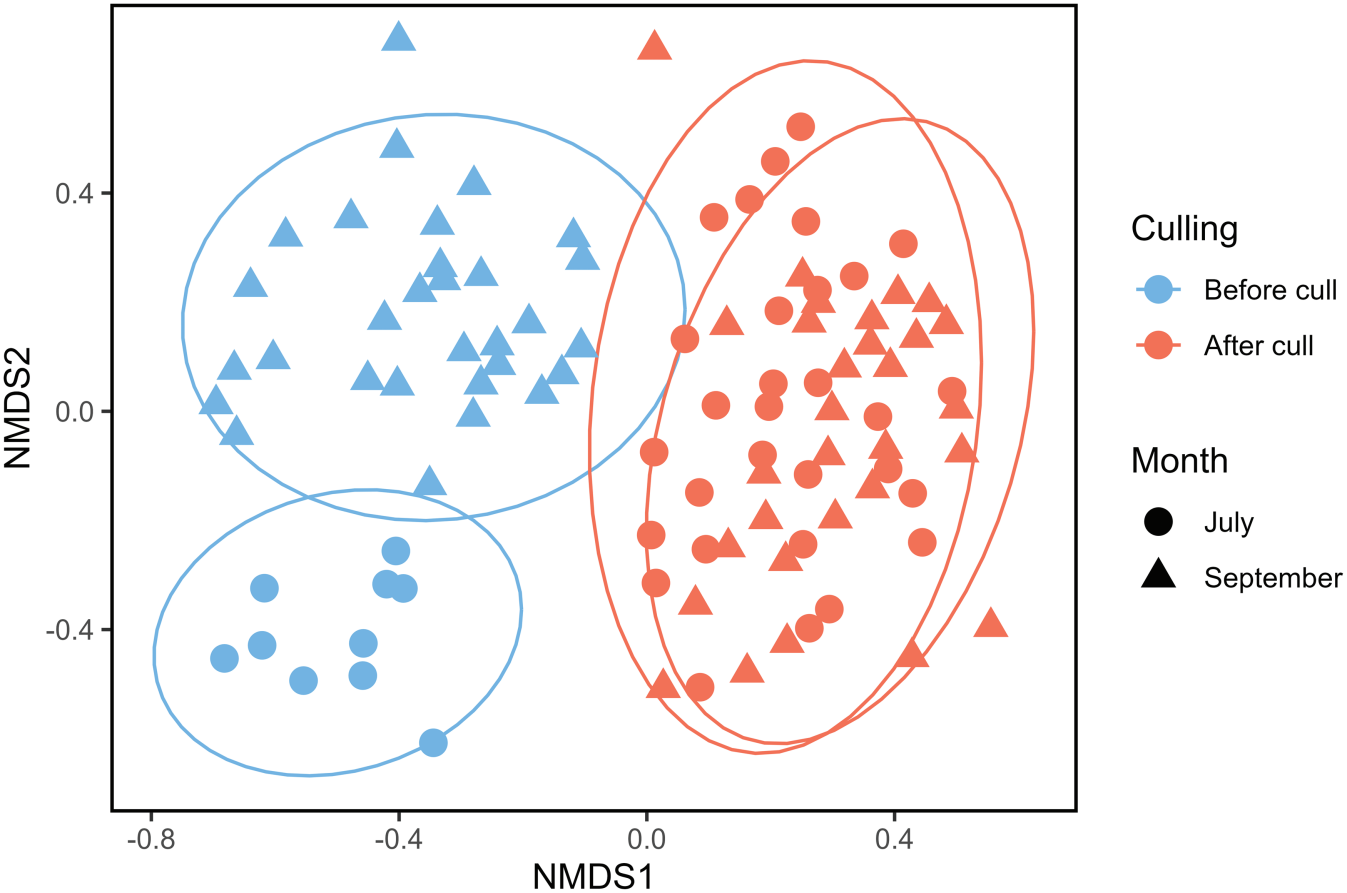
NMDS plot of beetle abundance community composition comparing July (circle) and September (triangle), before (blue) and after (red) culling.

In total, we collected 24 species from 4 beetle families (Carabidae, Scarabaeidae, Geotrupidae, and Silphidae). We found a significant interactive effect of culling and month on the beetle community composition (PERMANOVA: *F* = 14.739, *P* < 0.001). Distinct beetle communities were present in July and September before culling, but these communities became similar after culling (Fig. 1). We found the beetle communities were more even in July and September after culling compared to before culling (Table 1).

**Table 1.** Pielou’s evenness values for July and September before and after culling.

	July	September
Before cull	0.801	0.610
After cull	0.919	0.876

**Fig. 2.**
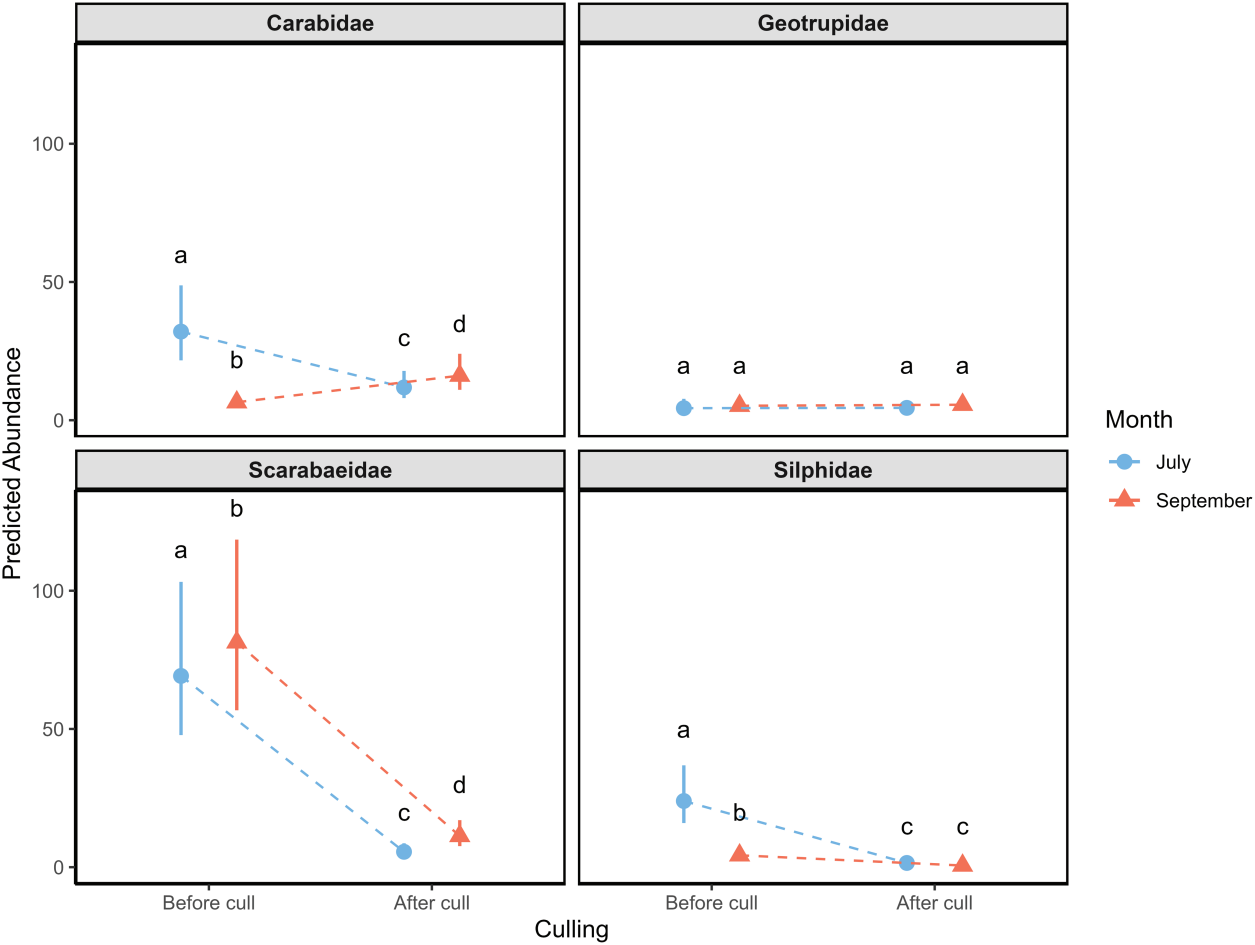
Predicted abundance of beetle community in July (blue) and September (red), and before and after deer culling, for each beetle family. Predicted abundances derived from GLMMs. Significance within each family is denoted by different letters (*P* < 0.05). Error bars represent 95% confidence intervals.

We found beetle families responded differently to culling and month (Fig. 2). Scarabaeidae had the largest loss in abundance after culling, but also displayed significantly reduced abundance in July compared to September, with this significant difference continuing after culling. Silphidae also had a significant reduction in abundance after culling, while culling had no effect on Geotrupidae abundance. Carabidae displayed mixed results, with culling causing both an increase and decrease in abundance, depending on the month.

**Fig. 3.**
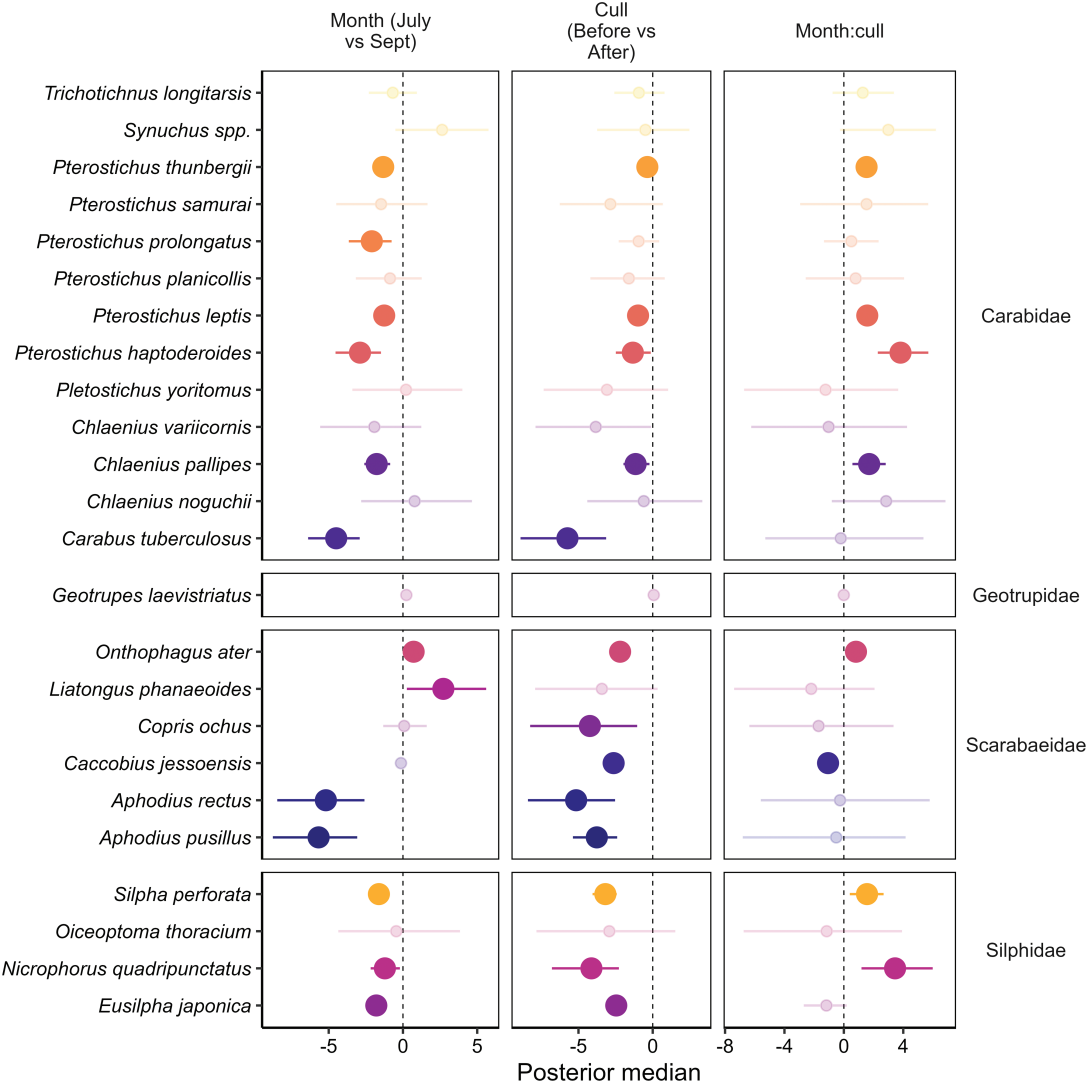
Bayesian ordination and regression analysis results of effect of season and cull on abundance of each beetle species. Credible intervals (95%) which do not cross the zero posterior median and displayed as not faded are significant. If significant (not faded), the side of the posterior median where the dot falls represent the direction of significance (e.g., for month, dots on the left that are not faded mean species had a significantly higher abundance in July compared to September). Species are grouped by family membership.

The multispecies Bayesian analysis revealed that only 2 species of Scarabaeidae had a significantly higher abundance in September compared to July (Fig. 3), while the remaining species of all families were evenly split between having a significantly higher abundance in July or no significant difference between seasons. For the culling effect, generally, the species that were significantly more abundant in July had a significantly higher abundance before culling than after culling. No species had a significantly higher abundance after the culling. Almost all Scarabaeidae species were significantly affected by the culling event, experiencing a reduction in abundance. Only some species had a significant interaction effect of both month and culling (see Supplementary Figure S1).

## Discussion

We investigated the effects of deer culling on ground-dwelling beetles in a unique island ecosystem. Our results suggest that the beetle community differed clearly between seasons, but after culling this seasonal difference was reduced. We also found that culling significantly reduced beetle abundance, but this was dependent on the beetle family and month. We found beetle community evenness increased after culling. Furthermore, we showed that approximately half of the beetle species we examined were impacted by culling, whereas the remaining species showed little change in abundance after culling. Notably, there were no beetle species that were higher in abundance after culling. Our study demonstrates a plausible link between ground-dwelling beetles and large herbivores on an island ecosystem and suggests there may be important ecological ramifications of reduced herbivore populations.

Each beetle family responded differently to the reduction of large herbivores. Scarabaeidae displayed the most dramatic loss in abundance after culling, which is likely linked to their dependence on dung as a food resource and breeding substrate (Nichols et al. 2009). Dung is generally a temporally consistent ephemeral resource, but with the loss in herbivore numbers on the island, dung availability was likely reduced (Butterworth et al. 2022). Therefore, species that are dependent on dung would be negatively affected. Silphidae also displayed a loss in abundance after culling, but to a lesser extent than Scarabaeidae. Silphidae are carrion beetles that are dependent on carrion for survival and reproduction, therefore the reduction in carcass availability due to the removal of herbivores from the ecosystem would directly impact Silphidae abundance (Holloway and Schnell 1997, Dekeirsschieter et al. 2011). Carrion is a patchy and unpredictable ephemeral resource, and those species dependent on carrion often have fluctuating populations, depending on carrion availability (Barton et al. 2019, Butterworth et al. 2022). This resource scarcity, potentially due to different deer mortality rates between seasons, is the likely reason for the seasonal variation in Silphidae abundance before culling observed in our study. Lastly, Carabidae both increased and decreased in abundance after culling, depending on the season. Carabidae are generally predators, though the family does occupy a wide range of ecological niches (Lövei and Sunderland 1996, Kotze et al. 2011). Some species of Carabidae are likely indirectly linked to herbivore populations due to changes in habitat structure due to herbivore grazing patterns (Ings and Hartley 1999). In our study system, the understorey vegetation has been diminished due to overgrazing (Yama et al. 2019). As herbivore numbers are reduced from culling, vegetation would recover (Donlan et al. 2002), thereby altering Carabidae habitat and prey abundance, resulting in mixed effects on Carabidae populations, depending on the individual species ecological niche.

Our study shows changes in the abundance of key groups of ground-dwelling beetles, most of which contribute to decomposition processes (Scarabaeidae, Geotrupidae, and Silphidae) (Nichols et al. 2009). The beetle communities became more even after culling, likely due to the reduction in abundance of the most dominant species. With the reduction of beetles following the culling of deer, there could be implications for the rate or efficiency of dung or carcass consumption and resulting nutrient turnover and dispersal pathways. It might also be that a broader suite of insects than assessed in this study could be impacted by the removal of large herbivores, such as flies and other insect species closely linked to decomposition (Braack 1987, Barton and Bump 2019, Benbow et al. 2019). We suggest that ongoing monitoring of populations of necrophagous species and necromass availability is needed to determine the longer-term effects of deer culling on the beetle community and their ecosystem services.

This study was limited to comparing only 1 year before and after the culling event, making it challenging to disentangle the effects of yearly variation (or other environmental variables) from the direct impact of culling. The study was also limited by the lack of a control as the whole island was subjected to the culling program. However, despite these limitations, this study still provides useful insight into the possible indirect effects of culling. To gain a comprehensive understanding of the long-term effects of culling, future research should encompass multiple years both before and after culling, as well as incorporate controls into the experimental design. This approach will enable the incorporation of yearly variations and facilitate a more accurate assessment of the communities’ ability to return to equilibrium after culling (Gordon et al. 2004). In particular, a more long-term approach may be able to identify if the reduction of the targeted herbivore leads to the eventual increase of herbivores that may have originally been competitively excluded.

While we observed the effects of deer culling on the abundance and composition of ground-dwelling beetles, it is probable that the extent of these changes depends on the intensity of culling. In our study system, there was a relatively high level of deer culling, which can be attributed to the clear results obtained. From a management standpoint, it is crucial to understand the degree to which changes in insect fauna can vary based on the level of culling, as well as to determine if there exists a threshold beyond which the loss of beetles will sharply escalate.

Our study emphasizes the need to understand comprehensively both the direct and indirect ecological impacts associated with reducing large herbivore species in an ecosystem. While culling can be an effective management tool to mitigate the effects of overabundant herbivores, future culling programs should carefully consider the complex ecological interactions between herbivore species and the broader ecosystem. By doing so, we can ensure the preservation of key ecological processes and maintain the integrity of food webs.

## Supplementary Material

Supplementary material is available at *Environmental Entomology* online.

nvae013_suppl_Supplementary_Figures_S1

## References

[CIT0001] Akashi N , Nakashizuka T. Effects of bark-stripping by sika deer (*Cervus nippon*) on population dynamics of a mixed forest in japan. Forest Ecol Manag. 1999:113(1):75–82. 10.1016/s0378-1127(98)00415-0

[CIT0002] Bardgett RD , Wardle DA. Herbivore-mediated linkages between aboveground and belowground communities. Ecology. 2003:84(9):2258–2268. 10.1890/02-0274

[CIT0003] Barton PS , Bump JK. Carrion decomposition. Springer International Publishing; 2019. p. 101–124.

[CIT0004] Barton PS , Cunningham SA, Lindenmayer DB, Manning AD. The role of carrion in maintaining biodiversity and ecological processes in terrestrial ecosystems. Oecologia. 2013:171(4):761–772. 10.1007/s00442-012-2460-3 23007807

[CIT0005] Barton PS , Evans MJ, Foster CN, Pechal JL, Bump JK, Quaggiotto M-M, Benbow ME. Towards quantifying carrion biomass in ecosystems. Trends Ecol Evol. 2019:34(10):950–961. 10.1016/j.tree.2019.06.001 31256926

[CIT0006] Barton PS , Manning AD, Gibb H, Wood JT, Lindenmayer DB, Cunningham SA. Experimental reduction of native vertebrate grazing and addition of logs benefit beetle diversity at multiple scales. J Appl Ecol. 2011:48(4):943–951. 10.1111/j.1365-2664.2011.01994.x

[CIT0007] Beguin J , Pothier D, Côté SD. Deer browsing and soil disturbance induce cascading effects on plant communities: a multilevel path analysis. Ecol Appl. 2011:21(2):439–451. 10.1890/09-2100.1 21563575

[CIT0008] Benbow ME , Barton PS, Ulyshen MD, Beasley JC, DeVault TL, Strickland MS, Tomberlin JK, Jordan HR, Pechal JL. Necrobiome framework for bridging decomposition ecology of autotrophically and heterotrophically derived organic matter. Ecol Monogr. 2019:89(1):e01331.

[CIT0009] Braack L. Community dynamics of carrion-attendant arthropods in tropical African woodland. Oecologia. 1987:72(3):402–409. 28311137 10.1007/BF00377571

[CIT0010] Brooks ME , Kristensen K, Van Benthem KJ, Magnusson A, Berg CW, Nielsen A, Skaug HJ, Machler M, Bolker BM. Glmmtmb balances speed and flexibility among packages for zero-inflated generalized linear mixed modeling. R J. 2017:9(2):378–400.

[CIT0011] Butterworth NJ , Benbow ME, Barton PS. The ephemeral resource patch concept. Biol Rev. 2022:98(3):697–726. 10.1111/brv.12926 36517934

[CIT0012] Cardinal E , Martin J-L, Côté SD. Large herbivore effects on songbirds in boreal forests: lessons from deer introduction on anticosti island. Écoscience. 2012:19(1):38–47. 10.2980/19-1-3441

[CIT0013] Côté SD , Rooney TP, Tremblay J-P, Dussault C, Waller DM. Ecological impacts of deer overabundance. Annu Rev Ecol Evol Syst. 2004:35(1):113–147. 10.1146/annurev.ecolsys.35.021103.105725

[CIT0014] Croft S , Franzetti B, Gill R, Massei G. Too many wild boar? Modelling fertility control and culling to reduce wild boar numbers in isolated populations. PLoS One. 2020:15(9):e0238429. 10.1371/journal.pone.0238429 32946480 PMC7500663

[CIT0015] Dekeirsschieter J , Verheggen F, Lognay G, Haubruge E. Large carrion beetles (coleoptera, Silphidae) in Western Europe: a review. Biotechnol Agron Soc Environ. 2011:15(3):435–447.

[CIT0016] Den Herder M , Virtanen R, Roininen H. Effects of reindeer browsing on tundra willow and its associated insect herbivores. J Appl Ecol. 2004:41(5):870–879. 10.1111/j.0021-8901.2004.00952.x

[CIT0017] Donlan CJ , Tershy BR, Croll DA. Islands and introduced herbivores: conservation action as ecosystem experimentation. J Appl Ecol. 2002:39(2):235–246. 10.1046/j.1365-2664.2002.00710.x

[CIT0018] Driscoll DA , Worboys GL, Allan H, Banks SC, Beeton NJ, Cherubin RC, Doherty TS, Finlayson CM, Green K, Hartley R, et al. Impacts of feral horses in the Australian alps and evidence-based solutions. Ecol Manag Restor. 2019:20(1):63–72.

[CIT0019] Englmeier J , Mitesser O, Benbow ME, Hothorn T, von Hoermann C, Benjamin C, Fricke U, Ganuza C, Haensel M, Redlich S, et al. Diverse effects of climate, land use, and insects on dung and carrion decomposition. Ecosystems. 2022:26:397–411.

[CIT0020] Enoki T , Yabe T, Koizumi T. Changes in spatial patterns of sika deer distribution and herbivory of planted seedlings: a comparison before and after deer population control by culling. J For Res. 2016:21(2):84–91. 10.1007/s10310-015-0515-0

[CIT0021] Eschtruth AK , Battles JJ. Acceleration of exotic plant invasion in a forested ecosystem by a generalist herbivore. Conserv Biol. 2009:23(2):388–399. 10.1111/j.1523-1739.2008.01122.x 19183209

[CIT0022] Foster CN , Barton PS, Lindenmayer DB. Effects of large native herbivores on other animals. J Appl Ecol. 2014:51(4):929–938. 10.1111/1365-2664.12268

[CIT0023] Gerhardt P , Arnold JM, Hackländer K, Hochbichler E. Determinants of deer impact in European forests—a systematic literature analysis. For Ecol Manage. 2013:310:173–186.

[CIT0024] Gill R , Beardall V. The impact of deer on woodlands: the effects of browsing and seed dispersal on vegetation structure and composition. For: Int J For Res. 2001:74(3):209–218.

[CIT0025] Gorchov DL , Blossey B, Averill KM, Dávalos A, Heberling JM, Jenkins MA, Kalisz S, McShea WJ, Morrison JA, Nuzzo V, et al. Differential and interacting impacts of invasive plants and white-tailed deer in eastern US forests. Biol Invasions. 2021:23(9):2711–2727. 10.1007/s10530-021-02551-2

[CIT0026] Gordon I , Snape M, Fletcher D, Howland B, Coulson G, Festa‐Bianchet M, Caley P, McIntyre S, Pople T, Wimpenny C, et al. Herbivore management for biodiversity conservation: a case study of kangaroos in the Australian Capital Territory (ACT). Ecol Manage Restor. 2021:22(S1):124–137.

[CIT0027] Gordon IJ , Hester AJ, Festa-Bianchet M. The management of wild large herbivores to meet economic, conservation and environmental objectives. J Appl Ecol. 2004:41(6):1021–1031.

[CIT0028] Holloway AK , Schnell GD. Relationship between numbers of the endangered American burying beetle *Nicrophorus americanus* Olivier (coleoptera: Silphidae) and available food resources. Biol Conserv. 1997:81(1–2):145–152. 10.1016/s0006-3207(96)00158-9

[CIT0029] Hui FK. Boral–Bayesian ordination and regression analysis of multivariate abundance data in R. Methods Ecol Evol. 2016:7(6):744–750. 10.1111/2041-210x.12514

[CIT0030] Iida T , Soga M, Koike S. Effects of an increase in population of sika deer on beetle communities in deciduous forests. ZooKeys. 2016:625:67–85. 10.3897/zookeys.625.9116 PMC5096363 27833427

[CIT0031] Ikeda T , Takahashi H, Igota H, Matsuura Y, Azumaya M, Yoshida T, Kaji K. Effects of culling intensity on diel and seasonal activity patterns of sika deer (*Cervus nippon*). Sci Rep. 2019:9(1):17205. 10.1038/s41598-019-53727-9 31748671 PMC6868152

[CIT0032] Ikeda T , Takahashi H, Yoshida T, Igota H, Kaji K. Evaluation of camera trap surveys for estimation of sika deer herd composition. Mamm Study. 2013:38(1):29–33. 10.3106/041.038.0103

[CIT0033] Ikeda T , Takahashi H, Yoshida T, Igota H, Matsuura Y, Takeshita K, Kaji K. Seasonal variation of activity pattern in sika deer (*Cervus nippon*) as assessed by camera trap survey. Mamm Study. 2015:40(4):199–205. 10.3106/041.040.0401

[CIT0034] Ings T , Hartley S. The effect of habitat structure on carabid communities during the regeneration of a native Scottish forest. Forest Ecol Manag. 1999:119(1–3):123–136. 10.1016/s0378-1127(98)00517-9

[CIT0035] Kaji K , Takahashi H, Tanaka J, Tanaka Y. Variation in the herd composition counts of sika deer. Popul Ecol. 2005:47(1):53–59. 10.1007/s10144-004-0200-1

[CIT0036] Kaji K , Takeshita K. Irruptive dynamics of sika deer: search for the mechanism. In: Kaji K, Uno H, Iijima H, editors. Sika deer: life history plasticity and management. Singapore: Springer; 2022. p. 309–326.

[CIT0037] Kotze DJ , Brandmayr P, Casale A, Dauffy-Richard E, Dekoninck W, Koivula M, Lovei G, Mossakowski D, Noordijk J, Paarmann W, et al. Forty years of carabid beetle research in Europe—from taxonomy, biology, ecology and population studies to bioindication, habitat assessment and conservation. ZooKeys. 2011:100:55–148. 10.3897/zookeys.100.1523 PMC3131012 21738408

[CIT0038] Lenth RV. emmeans: Estimated Marginal Means, aka Least-Squares Means. R package version 1.8.5; 2023. https://cran.r-project.org/web/packages/emmeans/index.html. (2023 March 28, date last accessed) .

[CIT0039] Lövei GL , Sunderland KD. Ecology and behavior of ground beetles (Coleoptera: Carabidae). Annu Rev Entomol. 1996:41(1):231–256. 10.1146/annurev.en.41.010196.001311 15012329

[CIT0040] Lüdecke D , Ben-Shachar M, Patil I, Waggoner P, Makowski D. Performance: an r package for assessment, comparison and testing of statistical models. J Open Source Softw. 2021:6(60):3139.

[CIT0041] Mahon MB , Fisk MC, Crist TO. Interactive effects of white-tailed deer, an invasive shrub, and exotic earthworms on leaf litter decomposition. Ecosystems. 2020:23(7):1523–1535. 10.1007/s10021-020-00485-9

[CIT0042] Nichols E , Gardner TA, Peres CA, Spector S. Co-declining mammals and dung beetles: an impending ecological cascade. Oikos. 2009:118(4):481–487. 10.1111/j.1600-0706.2008.17268.x

[CIT0043] Nichols E , Spector S, Louzada J, Larsen T, Amezquita S, Favila M, Network TSR. Ecological functions and ecosystem services provided by Scarabaeinae dung beetles. Biol Conserv. 2008:141(6):1461–1474.

[CIT0044] Nugent G , Choquenot D. Comparing cost-effectiveness of commercial harvesting, state-funded culling, and recreational deer hunting in New Zealand. Wildl Soc Bull. 2004:32(2):481–492. 10.2193/0091-7648(2004)32[481:ccochs]2.0.co;2

[CIT0045] Oksanen J , Simpson GL, Blanchet FG, Kindt R, Legendre P, Minchin PR, O’Hara RB, Solymos P, Stevens MHH, Szoecs E, et al. Vegan: Community ecology package. R package version 2.6-4; 2022. https://cran.r-project.org/web/packages/vegan/index.html. (2023 March 23, date last accessed).

[CIT0046] Putman R , Apollonio M, Andersen R. Ungulate management in Europe: problems and practices. England: Cambridge University Press; 2011.

[CIT0047] R Core Team. R: a language and environment for statistical computing. Vienna (Austria): R Foundation for Statistical Computing; 2023.

[CIT0048] Seki Y , Koganezawa M. Does sika deer overabundance exert cascading effects on the raccoon dog population? J Forest Res. 2013:18(1):121–127. 10.1007/s10310-011-0332-z

[CIT0049] Sharp RL , Larson LR, Green GT. Factors influencing public preferences for invasive alien species management. Biol Conserv. 2011:144(8):2097–2104. 10.1016/j.biocon.2011.04.032

[CIT0050] Suttiprapan P , Nakamura H. Species composition and seasonal abundance of carabid beetles by three sampling methods on the campus of the faculty of agriculture, Shinshu University. Jpn J Environ Entomol Zool. 2007:18(2):83–90.

[CIT0051] Tanentzap AJ , Kirby KJ, Goldberg E. Slow responses of ecosystems to reductions in deer (Cervidae) populations and strategies for achieving recovery. Forest Ecol Manag. 2012:264:159–166. 10.1016/j.foreco.2011.10.005

[CIT0052] Ueno S-L , Kurosawa Y, Sato M. The coleoptera of japan in color ii. Osaka Japan: Hoikusya. 1985.

[CIT0053] Velamazán M , San Miguel A, Escribano R, Perea R. Threatened woody flora as an ecological indicator of large herbivore introductions. Biodivers Conserv. 2017:26(4):917–930. 10.1007/s10531-016-1279-3

[CIT0054] Wäber K , Spencer J, Dolman PM. Achieving landscape-scale deer management for biodiversity conservation: The need to consider sources and sinks. J Wildl Manage. 2013:77(4):726–736.

[CIT0055] Wickham H. Ggplot2: Elegant graphics for data analysis. Switzerland: Springer-Verlag New York;. 2016.

[CIT0056] Yama H , Soga M, Evans MJ, Iida T, Koike S. The morphological changes of moths on Nakajima Island, Hokkaido, Japan. Environ Entomol. 2019:48(2):291–298. 10.1093/ee/nvz011 30810733

