# Supplementary material for: Drastic changes in ground-dwelling beetle communities following high-intensity deer culling: insights from an island ecosystem
Source: Environ Entomol. 2024 Feb 25;53(2):223–9. doi: 10.1093/ee/nvae013 (PMC11008735; doi:10.1093/ee/nvae013)
Supplement: nvae013_suppl_Supplementary_Figures_S1 [file nvae013_suppl_supplementary_figures_s1.docx]

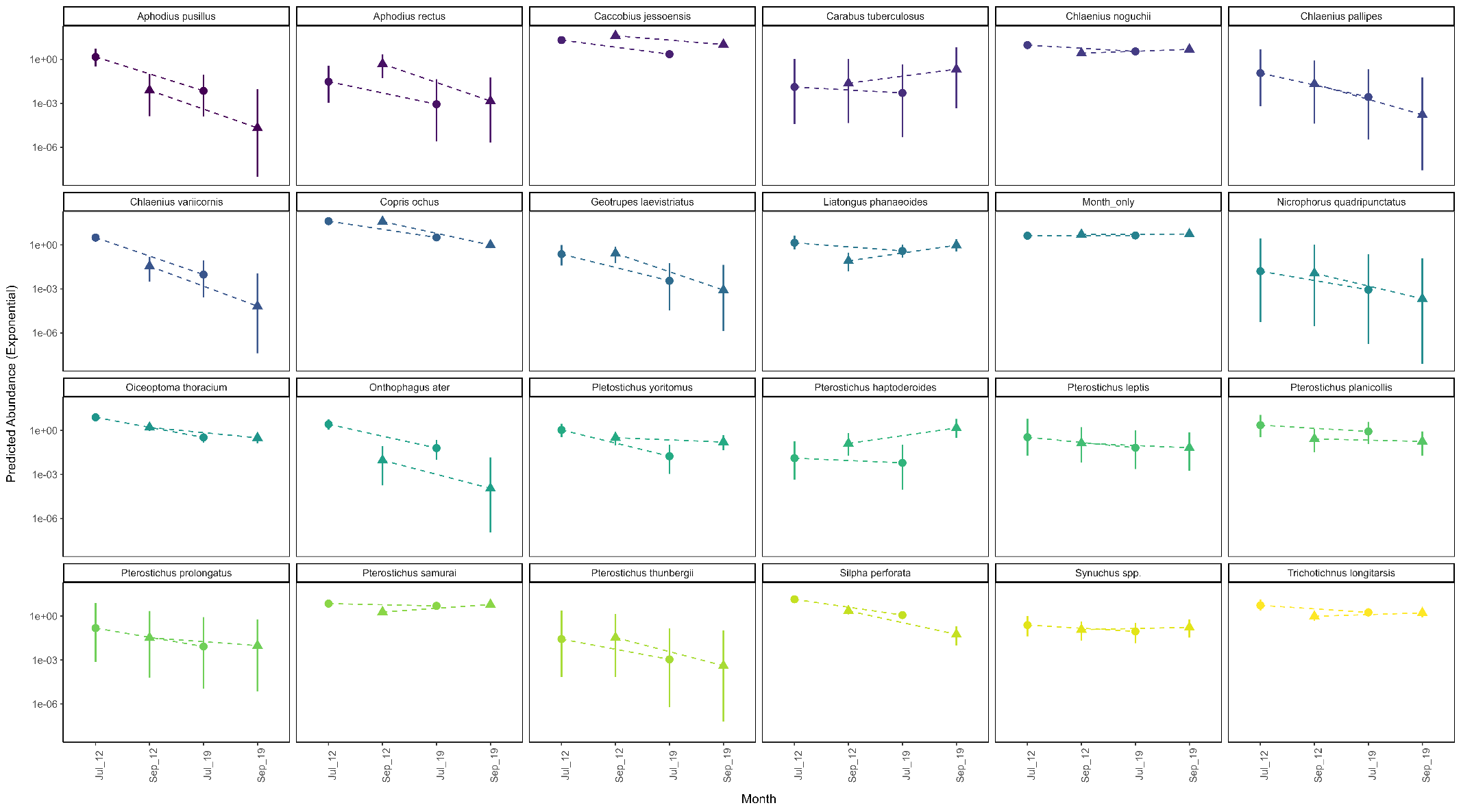


**Figure S1.** Prediction plot for each species based on the predictor variables extracted from the Bayesian analysis. Circles represent July, triangles represent September, with dashed line linking the same month before and after culling.
